# Characterisation and prognostic value of tertiary lymphoid structures in oral squamous cell carcinoma

**DOI:** 10.1186/1472-6890-14-38

**Published:** 2014-08-23

**Authors:** Anna M Wirsing, Oddveig G Rikardsen, Sonja E Steigen, Lars Uhlin-Hansen, Elin Hadler-Olsen

**Affiliations:** 1 Department of Medical Biology, Faculty of Health Sciences, University of Tromsø, Tromsø 9037, Norway; 2 Department of Otorhinolaryngology, University Hospital of North Norway, Tromsø 9038, Norway; 3 Diagnostic Clinic – Clinical Pathology, University Hospital of North Norway, Tromsø 9038, Norway

**Keywords:** Oral squamous cell carcinoma, Prognostic factor, Tertiary lymphoid structure, B-cell, High-endothelial venule, Follicular dendritic cell, Germinal centre

## Abstract

**Background:**

Oral squamous cell carcinomas are often heavily infiltrated by immune cells. The organization of B-cells, follicular dendritic cells, T-cells and high-endothelial venules into structures termed tertiary lymphoid structures have been detected in various types of cancer, where their presence is found to predict favourable outcome. The purpose of the present study was to evaluate the incidence of tertiary lymphoid structures in oral squamous cell carcinomas, and if present, analyse whether they were associated with clinical outcome.

**Methods:**

Tumour samples from 80 patients with oral squamous cell carcinoma were immunohistochemically stained for B-cells, follicular dendritic cells, T-cells, germinal centre B-cells and high-endothelial venules. Some samples were sectioned at multiple levels to assess whether the presence of tertiary lymphoid structures varied within the tumour.

**Results:**

Tumour-associated tertiary lymphoid structures were detected in 21 % of the tumours and were associated with lower disease-specific death. The presence of tertiary lymphoid structures varied within different levels of a tissue block.

**Conclusions:**

Tertiary lymphoid structure formation was found to be a positive prognostic factor for patients with oral squamous cell carcinoma. Increased knowledge about tertiary lymphoid structure formation in oral squamous cell carcinoma might help to develop and guide immune-modulatory cancer treatments.

## Background

Oral squamous cell carcinomas (OSCCs) are tumours known to metastasize to lymph nodes at an early stage of their development [1]. Despite current improvements in clinical management of this cancer type, mortality and morbidity rates of OSCC patients have remained high over the last decades, with an average 5-year survival rate of about 50% [2,3]. The TNM staging of the tumour, and especially the presence and extent of lymph node metastasis (N stage), have considerable prognostic importance for patients with OSCC [4] and are used to guide treatment strategies. However, tumours of the same clinical stage may respond differently to the same treatment and may also have distinct clinical outcomes [5].

Considerable interest has been devoted to the complex interplay between tumour cells and host-immune response, and especially to how infiltrating immune cells might affect the clinical outcome of cancer patients. Anti-tumour functions of tumour-infiltrating lymphocytes (TILs), particularly of T-cells, have been observed in numerous types of cancer [6]. Accumulating evidence indicates that infiltrating immune cells may also be involved in the development and progression of oral cancer, where they have shown both favourable and detrimental effects [7]. It is well established that immune cells infiltrating to sites of chronic inflammation organize themselves both anatomically and functionally similar to secondary lymphoid organs (SLOs), a phenomenon called tertiary lymphoid structure (TLS) formation [8]. Similar to lymphoid follicles, TLSs typically comprise aggregates of B-cells in a meshwork of follicular dendritic cells (FDCs) that are then surrounded by T-cells as well as specialized blood vessels referred to as high-endothelial venules (HEVs) [9]. HEVs express the lymphoid chemokine peripheral node addressin (PNAd), which binds to L-Selectin on naive lymphocytes and thus promote lymphocyte recruitment to sites of chronic inflammation [10]. Furthermore, a complex interplay between different lymphotoxin- and chemokine-induced signalling pathways is required for the initiation of TLS formation [9]. In contrast to lymph nodes, TLSs are not encapsulated, resulting in constitutive, direct antigenic stimulation from their surrounding microenvironment [11]. Lymphatic vessels have also been found in association with TLSs, but their functional interplay is not yet fully clarified [12]. The presence of ongoing germinal centre (GC) reactions in B-cell clusters of ectopic lymphoid structures has been reported, indicating that adaptive immunity can be triggered at sites different from SLOs [11]. In autoimmune disorders, formation of ectopic lymphoid tissue is associated with disease progression [11], whereas TLS development in breast, ovarian, non-small-cell lung, renal and colorectal cancer is reported to be associated with a favourable prognosis [13-23].

The aim of the present study was to evaluate the incidence of TLSs in OSCCs, and if present, analyse whether they were associated with clinical outcome. The study included tissue samples and clinical data from 80 patients diagnosed with OSCCs between 1986 and 2002 at the Diagnostic Clinic – Clinical Pathology, University Hospital of North Norway (UNN). The presence of TLSs was determined based on immunohistochemical staining patterns of B-cells, FDCs, GC B-cells, T-cells and HEVs. We established that the presence of TLSs is a positive prognostic factor for patients with OSCC. Understanding and interpreting TLS formation in OSCC might help to implement and guide immunotherapeutic interventions. In terms of individual clinical management, reliable prognostic markers together with targeted anticancer therapies might improve the consistently low survival rates in patients with oral cancer.

## Methods

### Patients

The study broadly follows the REMARK recommendations for tumour marker prognostic studies [24]. Eighty patients with histologically verified primary SCC of the oral cavity in the period 1986–2002 were selected from the archives of the Diagnostic Clinic – Clinical Pathology, UNN. The last day of follow-up was January 1st, 2012. The specimens were formalin-fixed, paraffin-embedded tumour resections or biopsies from the mobile tongue, floor of the mouth, buccal mucosa, gingiva and soft and hard palate. We excluded specimens from the base of the tongue and the tonsils – sites naturally rich in lymphatic tissue. Patients with a history of former head and neck cancer were also excluded from the study. Clinical data, including tumour staging according to the TNM-classification and treatment modalities, were retrieved from the patients’ hospital files, pathology reports and the Statistics of Norway, Cause of Death Registry, and are listed in Table 1. Information on the HPV status determined by p16 immunohistochemical staining was obtained from the Diagnostic Clinic – Clinical Pathology, UNN, and is also presented in Table 1. In addition to the patient samples, formalin-fixed, paraffin-embedded normal oral tissue was used as control. The study was approved by the Regional Committee for Medical and Health Research Ethics, Northern Norway (REK-number 22/2007), which also gave the permission to access patient files containing the clinical data. All clinical data were kept anonymous.

**Table 1:** Comparison of clinicopathological variables between 80 OSCC patients with and without TLSs using Pearson’s Chi-square test

	**TLS-negative**	**TLS-positive**	**P-value**
	**(N = 63) (no. (%))**	**(N = 17) (no. (%))**	
**Gender**			
Male	35 (55.6)	11 (64.7)	0.498
Female	28 (44.4)	6 (35.3)	
**Age at diagnosis, years**			
Mean	63.19	63.71	0.178
0-59	23 (36.5)	6 (35.3)	0.926
≥ 60	40 (63.5)	11 (64.7)	
**Smoking history**			
Never smoker	14 (22.2)	4 (23.5)	
Former smoker	10 (15.9)	1 (5.9)	0.722
Current smoker	34 (54.0)	11 (64.7)	
Unknown	5 (7.9)	1 (5.9)	
**Alcohol consumption**			
Never	12 (19.0)	1 (5.9)	
≤ 1 times weekly	24 (38.1)	6 (35.3)	0.114
> 1 times weekly or daily	17 (27.0)	3 (17.6)	
Unknown	10 (15.9)	7 (41.2)	
**Tumour site**			
Mobile tongue	29 (46.0)	9 (52.9)	
Floor of mouth	17 (27.0)	5 (29.4)	
Soft palate	1 (1.6)	0 (0.0)	0.956
Buccal mucosa	7 (11.1)	1 (5.9)	
Alveolar ridge	8 (12.7)	2 (11.8)	
Unknown	1 (1.6)	0 (0.0)	
**Tumour differentiation**			
Well	20 (31.7)	10 (58.8)	
Moderate	39 (61.9)	5 (29.4)	0.058
Poor	4 (6.3)	2 (11.8)	
**T stage**			
T1	23 (36.5)	6 (35.3)	
T2	18 (28.6)	9 (52.9)	0.187
T3, T4	21 (33.3)	2 (11.8)	
Unknown	1 (1.6)	0 (0.0)	
**N stage**			
N0	41 (65.1)	13 (76.5)	
			0.670
N+	17 (27.0)	3 (17.6)	
Unknown	5 (7.9)	1 (5.9)	
**M stage**			
M0	57 (90.5)	17 (100.0)	
M+	1 (1.6)	0 (0.0)	0.417
Unknown	5 (7.9)	0 (0.0)	
**Treatment**			
Surgery local +/− neck resection	7 (11.1)	2(11.8)	
Surgery and radiotherapy	41 (65.1)	12 (70.6)	
Radiotherapy +/− chemotherapy	8 (12.7)	3 (17.6)	0.700
None or palliative	5 (7.9)	0 (0.0)	
Unknown	2 (3.2)	0 (0.0)	
**HPV/p16**			
Negative	52 (82.5)	16 (94.1)	
Positive	5 (7.9)	1 (5.9)	0.386
Unknown	6 (9.5)	0 (0.0)	

### Immunohistochemistry

Four-micrometer-thick sections of formalin-fixed, paraffin-embedded tissue of patients with OSCC on Superfrost Plus slides were subjected to immunohistochemical staining. From patients where several tumour-containing paraffin-blocks were available, a block with representative material, based on H/E staining, was chosen without specific evaluation of the inflammatory infiltrate. Before staining, all specimens were incubated overnight at 60°C, deparaffinised in xylene, rehydrated in graded alcohol baths and subjected to heat-induced antigen retrieval in 0.01 M sodium citrate buffer at pH 6.0. Prior to antibody incubation, inherent peroxidase activity in the tissue was blocked with 3% H_2_O_2_ (Ventana Medical Systems, France or Dako Glostrup, Denmark). The following primary antibodies were used: Mouse anti-CD20, clone L26; Mouse anti-CD21, clone 2G9; Mouse anti-bcl-6, clone GI191E/A8; Mouse anit-CD34, clone QBEnd/10; Rabbit anti-CD3, clone 2GV6 (all from Ventana Medical Systems, France); Mouse anti-Podoplanin, clone D2-40 (Dako, Glostrup, Denmark) and Rat anti-PNAd, clone MECA-79, (Biolegend, San Diego). Dilutions and incubation times are listed in Table 2. Except for the PNAd antibody, all immunohistochemical stainings were done in the automated slide stainer Ventana Benchmark, XT (Ventana) at the Diagnostic Clinic – Clinical Pathology, UNN, which is accredited according to the ISO/IEC 15189 standard for the respective stainings, using the same protocols, positive and negative controls as in the clinical routines. For these automated stainings, a cocktail of HRP labelled goat anti-mouse IgG/IgM and mouse anti-rabbit secondary antibodies together with diaminobenzidine from the Ventana UltraView Universal DAB Detection Kit (#760-500, Ventana) were applied for visualization.

**Table 2:** Antibodies for immunohistochemistry

**Antibody**	**Dilution**	**Incubation time**
Mouse anti-CD20, clone L26, Ventana Medical Systems, France	Pre-diluted	16 min
Mouse anti-CD21, clone 2G9, Ventana	Pre-diluted	32 min
Mouse anti-bcl-6, clone GI191E/A8, Ventana	Pre-diluted	40 min
Mouse anti-Podoplanin, clone D2-40, Dako, Glostrup, Denmark	1:25	32 min
Mouse anit-CD34, clone QBEnd/10, Ventana	Pre-diluted	32 min
Rabbit anti-CD3, clone 2GV6, Ventana	Pre-diluted	16 min
Rat anti-PNAd, clone MECA-79, Biolegend, San Diego	1:25	30 min
Goat anti-rat light chain antibody, #AP202P, Millipore, Temecula, CA	1:250	30 min

Manual staining with the PNAd primary antibody was performed as previously described [25], using HRP-labelled goat anti-rat light chain secondary antibody (#AP202P, Millipore, Temecula, CA) and diaminobenzidine (Dako EnVision + System-Horseradish Peroxidase, Dako) for detection. Counterstaining was done with Harris hematoxylin (Sigma-Aldrich, St. Louis, MO). Finally, the sections were dehydrated in graded alcohol and xylene baths, and mounted with Histokit (Chemi-teknikk, Oslo, Norway). Negative controls were treated identically but with the primary antibodies replaced by the antibody diluting solution. Formalin-fixed, paraffin-embedded human lymph nodes served as positive controls for the PNAd staining. Negative control sections never showed any staining, whereas the positive control sections (lymph nodes) always showed positive staining confined to the cells that were supposed to be positive (data not shown). The specificity of the PNAd antibody was evaluated on consecutive sections from six different OSCC samples and three samples of normal oral mucosa. These OSCC and normal tissue sections were assessed for overlapping immunohistochemical staining for the PNAd antibody, the blood vessel marker CD34 and the lymphatic endothelial cell marker D2-40. In the OSCC samples, sporadic CD34+ blood vessels were to a minor degree positive for PNAd, whereas no D2-40+ lymphatic vessels were positive, indicating a high degree of antibody specificity. No HEV staining was seen in the three samples from normal oral mucosa.

### Immunohistochemical evaluation

Eighty patients were included in the study. In 25 of the patients, the presence of TLSs was evaluated at a single level in the tumour tissue block. In 45 of the patients – randomly chosen from the 80 patients – TLS formation was evaluated at two discrete levels at about 100 μm distance in the tissue block. Additionally, tumour tissue blocks from 10 of the patients – nine of them negative for TLSs in the superficial level – were cut down completely and presence of TLSs was evaluated at 100 μm distance throughout the tumour sample.

We used a two-step method for TLS detection. First, the tissue sections were immunohistochemically stained for the pan B-cell marker CD20 and assigned to three different groups based on their staining pattern: obvious B-cell aggregates, indistinct aggregates of B-cells and no or scattered B-cells. Second, staining for the FDC marker CD21, the T-cell marker CD3 and the HEV marker PNAd was performed on consecutive sections of those with obvious and indistinct B-cell aggregates. For FDC evaluation, areas with clusters of B-cells were examined at high-power magnification (400×). All tumours that had one or several accumulations of B-cells containing CD21 positive FDCs were defined as TLS-positive. All TLSs also contained HEVs and T-cells. The TLS-positive tumours were further subdivided into classical and non-classical TLSs. A classical TLS was defined as a B-cell aggregate containing a continuous FDC meshwork, and a non-classical TLS as a B-cell aggregate with a more diffuse distribution of the FDCs. Sections from seven of the TLS-positive tumours were stained with BCL-6 to verify the presence of GC B-cells in B-cell clusters of TLSs.

### Statistical analysis

All statistical analyses were performed with the SPSS software version 22.0 for Windows (IBM, Armonk, NY). The association between various clinicopathological variables was examined by the Pearson’s Chi-square test. Disease-specific death (DSD) and disease-specific survival (DSS) curves were estimated in univariate analyses and by Kaplan Meier method. The log-rank test was used to evaluate significant differences between the groups of patients. Variables that were statistically significant in the univariate analysis were entered into multivariate Cox regression analyses to identify independent prognostic factors in the presence of other variables. Validity of the proportionality assumption was verified by plotting log-minus-log plots. P-values less than 0.05 were considered statistically significant.

## Results

### Presence of TLSs in OSCC

**Figure 1:**
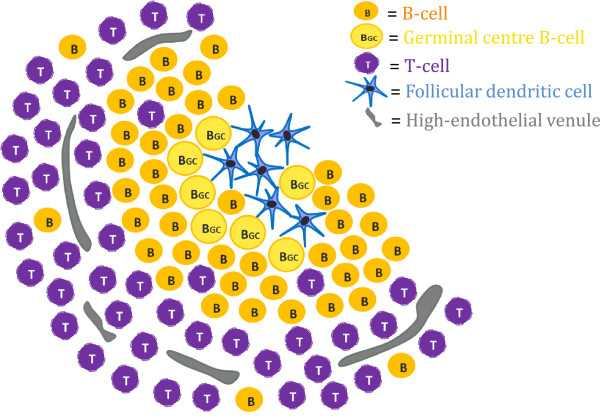
**Schematic model of tertiary lymphoid structures.** Specialized cell populations arrange themselves into distinct patterns forming a classical tertiary lymphoid structure (TLS).

**Figure 2:**
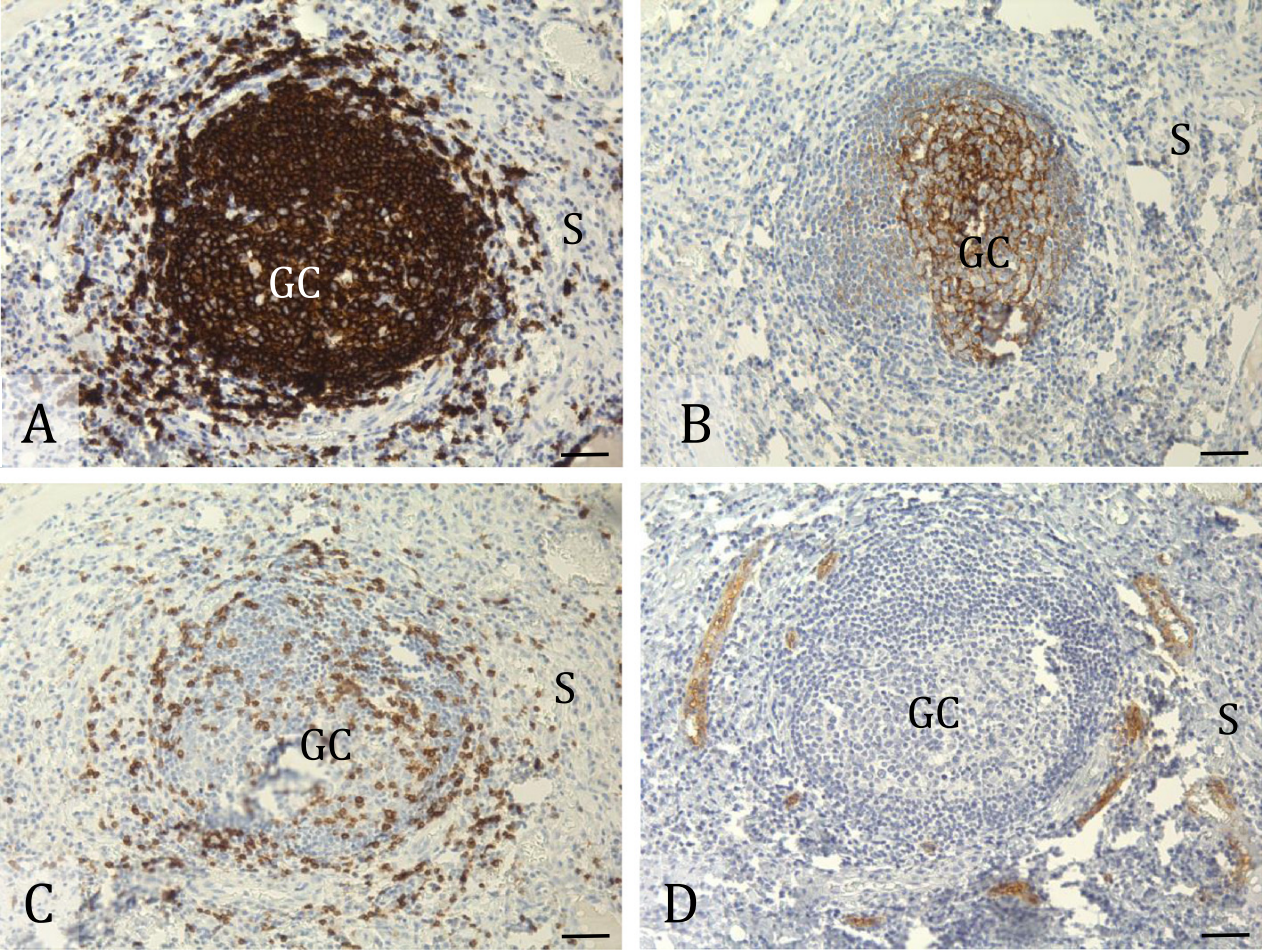
**Tertiary lymphoid structures in oral squamous cell carcinoma.** The pictures show representative immunohistochemical stainings on consecutive sections of the same oral squamous cell carcinoma (OSCC) tissue sample for detection of classical tertiary lymphoid structures (TLSs). A section that presents clusters of CD20+ B-cells **(A)** typically shows organized accumulations of follicular dendritic cells (FDCs) in a consecutive section stained for CD21 **(B)**. T-cell areas within and around the B-cell follicle are found by staining another consecutive section for CD3 **(C)**. High-endothelial venules (HEVs) adjacent to the B-cell follicle are detected when staining a consecutive section for PNAd, as shown in **(D)**. CD20+, CD21+ and CD3+ cells as well as PNAd + vessels are stained brown, and cell nuclei are stained blue by hematoxylin. Germinal centres are labelled “GC” and stroma surrounding the TLS is labelled “S” in the micrographs. Scale bars indicate 40 μm.

TLSs are highly organized structures that typically appear as clusters of B-cells containing FDCs. These clusters are then surrounded by T-cells and HEVs as shown schematically in Figure 1. We investigated the presence of TLSs in tumour specimens from 80 patients with OSCCs using immunohistochemistry. Sections with distinct or more diffuse B-cell aggregates were considered likely to have TLSs, and their consecutive sections were stained for FDCs, T-cells and HEVs, whereas sections without B-cell aggregates were not further analysed. At the first level assessed, TLSs were found in 13 of the 80 specimens. Eleven of these TLSs were found in sections with distinct B-cell aggregates, and only two in sections with diffuse B-cell aggregates. Pictures of a classical TLS are shown in Figure 2. One more TLS-positive tumour was identified by staining for TLSs at an additional level about 100 μm deeper in the tissue blocks from 45 of the patients. Three additional TLS-positive tumours were detected by assessing the whole tissue sample from 10 patients. These three TLS-positive tumours showed TLSs at multiple levels. Altogether, TLSs were found in 17 (21 %) of the 80 patients included in the study. The maximum number of TLSs in a single section was four, but usually not more than two TLSs were detected in each of the positive sections. The TLSs were mainly found in the peri-tumoural stroma within 0.5 mm distance from the tumour front, in lymphocyte-rich subepithelial areas.

**Figure 3:**
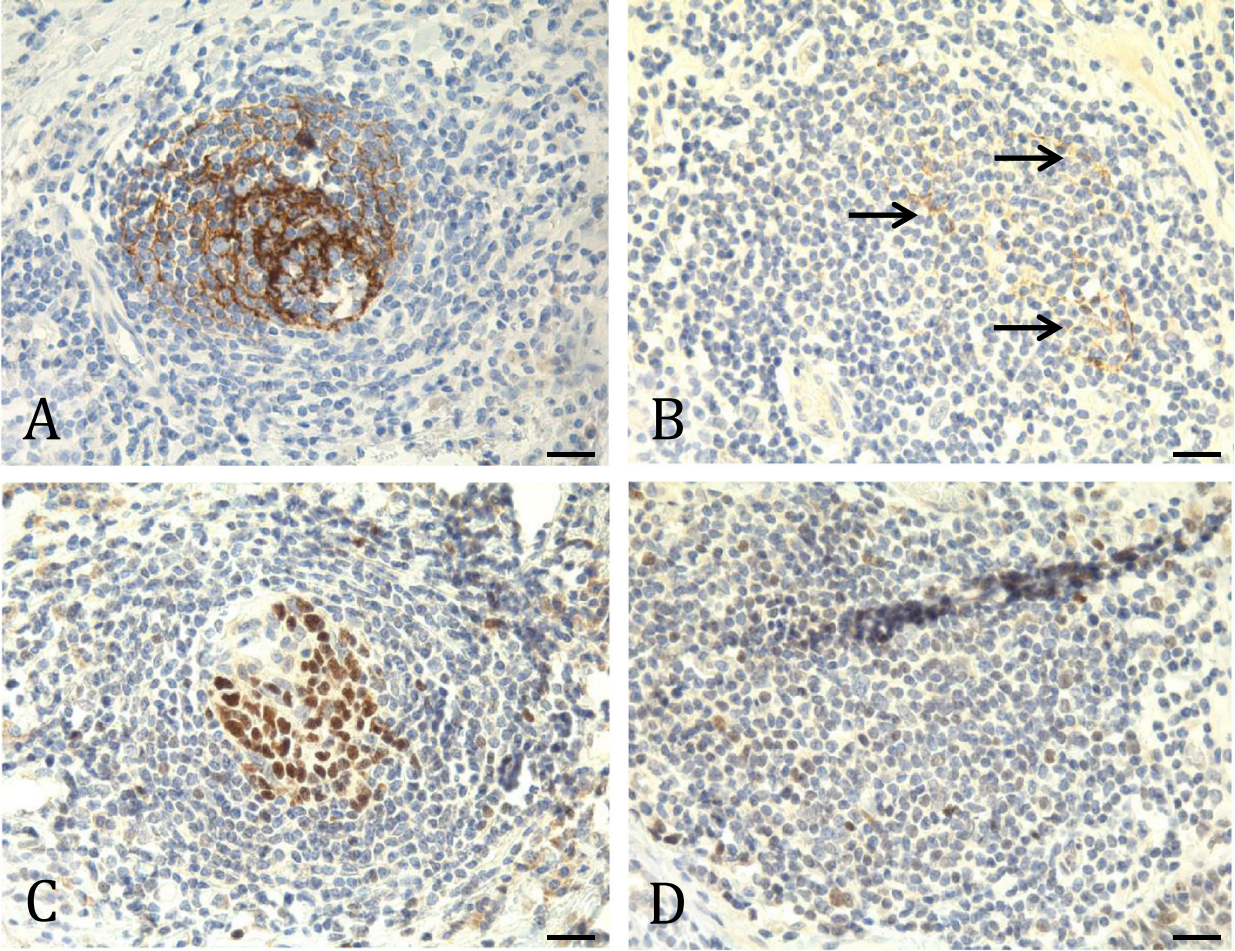
**Classical and non-classical tertiary lymphoid structures.** The pictures show representative immunohistochemical stainings on consecutive sections of two different oral squamous cell carcinoma (OSCC) tissue samples (A/C *vs.* B/D) for detection of classical (A/C) and non-classical (B/D) tertiary lymphoid structures (TLSs). B-cell follicles of classical TLSs normally comprise contiguous meshworks of CD21+ follicular dendritic cells (FDCs), as indicated in **(A)**, and show distinct accumulations of germinal centre (GC) B-cells when staining a consecutive section for BCL6, as presented in **(C)**. B-cell follicles of non-classical TLSs usually contain scattered FDCs, as shown in (**B**; arrows), and lack GC B-cells on a consecutive section stained for BCL6 **(D)**. In some cases, non-classical TLSs also show abnormal GCs with BCL6+ cells dispersed throughout the follicle (data not shown). CD21+ and BCL6+ cells are stained brown, and cell nuclei are stained blue by hematoxylin. Scale bars indicate 40 μm.

Within the B-cell aggregates, FDCs were found in either of two patterns: distinct meshworks (Figure 3A) or diffuse accumulations (Figure 3B) of CD21 positive cells. Only B-cell aggregates with contiguous FDC meshworks showed distinct accumulations of BCL6+ GC B-cells (Figure 3C) and are here referred to as *classical TLSs*. In the B-cell aggregates with diffuse accumulations of FDCs, GC B-cells were either absent (Figure 3D) or dispersed throughout the follicle, and these are here referred to as *non-classical TLSs*. Sometimes both classical and non-classical TLSs were found in the same section. Analyses of multiple tissue levels showed that some TLSs classified as non-classical on one tissue level presented a classical pattern on another tissue level and vice versa.

### Clinicopathological characteristics and prognostic value of TLSs

**Figure 4:**
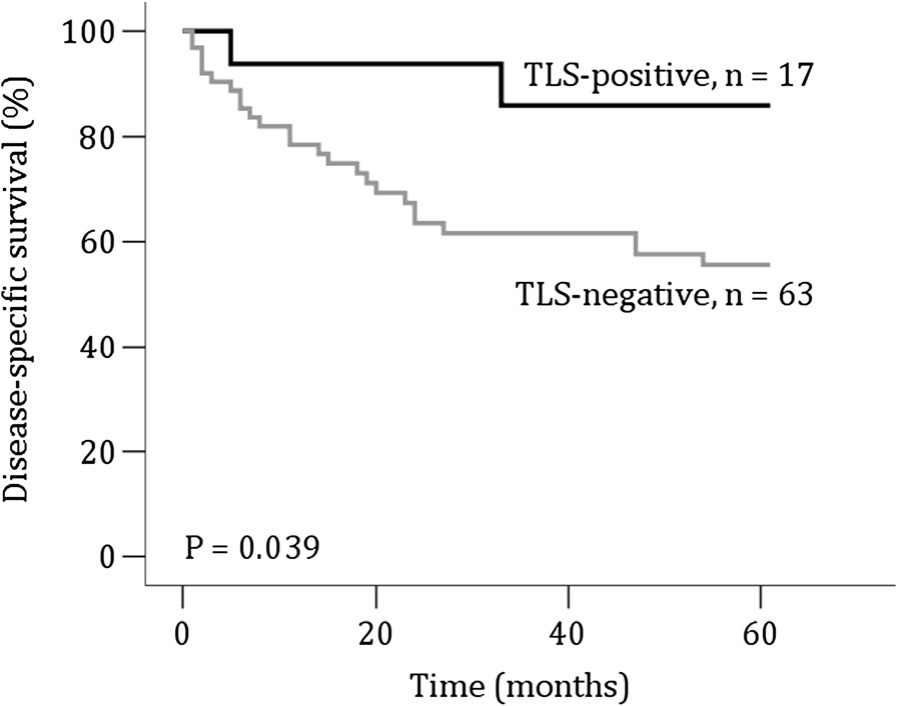
**Results from multiple level analysis: Kaplan Meier analysis of 5-year disease-specific survival for 80 patients with oral squamous cell carcinoma with and without tertiary lymphoid structures.** The presence of tertiary lymphoid structures (TLSs) is associated with improved survival in patients with oral squamous cell carcinoma (OSCC) (P = 0.039). The Kaplan-Meier curve shows a 5-year disease-specific survival rate of 88.2% for TLS-positive patients and 60.3% for TLS-negative patients. The P-value was calculated using the log-rank test.

Clinicopathological data of the patients were analysed for correlation with the presence of TLSs, and the results are presented in Table 1. Although not statistically significant, the majority of TLSs were found in patients with well-differentiated tumours. Further, there were more TLSs in T1 and T2 tumours compared to T3/T4 tumours. TLSs showed no statistically significant association with the other variables examined. The prognostic value of various clinicopathological variables in OSCC was investigated in univariate analysis using the log-rank test (Table 3). Based on the assessment of one tissue level, TLS-positive tumours indicated a trend toward improved survival. When the assessment of TLSs was based on multiple tissue levels, a significant association between the presence of TLSs and favourable outcome in OSSC patients was found, as shown in Figure 4. As patients presented various TLS subtypes (either classical, non-classical or both classical and non-classical), we analysed whether the TLS subtype influenced 5-year DSD. As presented in Additional file 1: Table S1, there was a tendency towards lower 5-year DSD for all patients with TLSs, regardless of the subtype. However, the differences were not statistically significant. Presence of classical TLSs alone or in combination with non-classical TLSs seemed to be associated with better prognosis compared to the presence of only non-classical TLSs, but again, no statistical significant difference between the subtypes was found (P = 0.304; data not shown). Furthermore, our results also confirmed the prognostic value of the T, N and M stages. The variables that showed statistically significant association with DSD in the univariate analyses (T, N stage and TLS) were entered into multivariate Cox regression analyses. The M status was excluded from multivariate analyses as there was only one M + patient. Proportional hazards assumptions were satisfied for multivariate analyses as shown by parallel curves for different categories of prognostic variables on log-minus-log plots (Additional file 1: Figure S1). In multivariate analyses, only the T status remained independently associated with disease-specific death (P < 0.001, Table 4).

**Table 3:** Clinicopathologic variables as predictors for 5-year disease-specific death in univariate analysis for 80 patients with OSCC

	**Patients (N = 80)**	**5-Year death (%)**	**P-value**
	**(no. (%))**		
**Gender**			
Male	46 (57.5)	37.0	0.403
Female	34 (42.5)	29.4	
**Age at diagnosis, years**			
0-59	29 (36.3)	31.0	0.637
≥ 60	51 (63.8)	35.3	
**Smoking history**			
Never smoker	18 (22.5)	27.8	
Former smoker	11 (13.8)	27.3	0.897
Current smoker	45 (56.3)	37.3	
Unknown	6 (7.5)	33.3	
**Alcohol consumption**			
Never	13 (16.3)	23.1	
≤ 1 times weekly	30 (37.5)	33.3	0.633
> 1 times weekly or daily	20 (25.0)	35.0	
Unknown	17 (21.3)	41.2	
**Tumour site**			
Mobile tongue	38 (47.5)	21.1	
Floor of mouth	22 (27.5)	40.9	0.074
All others*	20 (25.0)	50.0	
**Tumour differentiation**			
Well	30 (37.5)	26.7	
Moderate	44 (55.0)	36.4	0.296
Poor	6 (7.5)	50.0	
T stage**			
T1	29 (36.7)	20.7	
T2	27 (34.2)	11.1	<0.001
T3, T4	23 (29.1)	78.3	
**N stage**			
N0	54 (67.5)	22.2	
N+	20 (25.0)	70.0	<0.001
Unknown	6 (7.5)	16.7	
**M stage**			
M0	74 (92.5)	33.8	
M+	1 (1.3)	100.0	0.021
Unknown	5 (6.3)	20.0	
**HPV/p16**			
Negative	68 (85.0)	35.3	
Positive	6 (7.5)	16.7	0.720
Unknown	6 (7.5)	33.3	
**TLS single level**			
Negative	67 (83.8)	37.3	0.156
Positive	13 (16.3)	15.4	
**TLS multiple level**			
Negative	63 (78.8)	39.7	0.039
Positive	17 (21.3)	11.8	

P-values were calculated using the log-rank test.

*For univariate survival analysis, the tumour sites were grouped into three categories.

**Only 79 patients were analysed because the unknown case was taken out from the calculations.

**Table 4:** Results from multiple level analysis: multivariate analysis of 5-year disease-specific death according to Cox’s proportional hazards model*

**Variable**	**Hazard ratio**	**95% C.I.**	**P-value**
T stage	---	---	< 0.001
T stage (1) (T1 [n = 29] v. T2 [n = 27])	0.538	0.134 - 2.151	0.381
T stage (2) (T1 [n = 29] v. T3/T4 [n = 23])	7.237	2.814 - 18.612	< 0.001
N stage	---	---	0.359
N stage (1) (N0 [n = 54] v. N + [n = 20])	1.820	0.742 - 4.461	0.191
N stage (2) (N0 [n = 54] v. unknown [n = 5])	2.342	0.290 - 18.900	0.424
TLS (negative [n = 62] v. positive [n = 17])	2.409	0.556 - 10.448	0.240

*Only 79 patients were analysed because the case with unknown T stage was taken out from the calculations.

## Discussion

In the present study, we have demonstrated TLSs in OSCC by immunohistochemical analyses. To the best of our knowledge, this is the first report of TLSs in OSCCs. TLSs were found in 16% of the patients when a single level of the tumour was assessed, and in 21% of the patients when multiple levels of the tumours were analysed. This is a rather low occurrence compared to what has previously been reported in colorectal cancer and lung cancer, suggesting that the occurrence of TLSs varies among different types of tumours [14,19,26]. When assessed at a single level, presence of TLSs was not a significant predictor of survival. However, when analysed at multiple levels, their presence in the tumour was a positive prognostic factor. This indicates that the prognostic value of TLSs depends on the type of analysis, probably due to their rather infrequent occurrence and tumour heterogeneity. In multivariate analyses, only T stage turned out to be an independent prognostic factor. TLS status, however, performed better than N stage, which is recognized as one of the best prognostic factors in OSCCs [4]. In the TLS-positive tumours, either single or multiple TLSs were found in the same tissue section. In some of the TLSs, GC B-cells and FDC meshworks were observed, providing evidence that the TLSs comprised all cells needed to generate a functional immune response. We called lymphoid structures with defined FDC meshworks and GCs *classical TLSs*, as this phenotype has been mostly described for TLSs in literature. Besides the classical TLSs, we also found TLSs with diffuse accumulations of FDCs and scattered or absent GC B-cells that we termed *non-classical TLSs*. It remains elusive whether non-classical TLSs have the same immunological properties as classical TLSs. Immunohistochemistry on multiple tissue planes of the same tumour showed in some cases that classical and non-classical phenotypes corresponded to the same ectopic lymphoid structure. This implies that the two different patterns may be artefacts of the methodical approach of TLS detection. This is also supported by the fact that both classical and non-classical TLSs were found on the same tissue plane. Moreover, patients with TLSs showed prolonged survival regardless of TLS subtype, indicating that none of the TLS subtypes alone are particularly associated with survival. However, we found a trend towards better prognosis for patients with classical TLSs or with both classical and non-classical TLSs compared to patients with non-classical TLSs only. This indicates that, in some cases, non-classical TLSs could also represent immature follicles that may later develop into classical TLSs with full immunogenic properties. Previous studies have already reported the presence of fully and not fully mature TLSs in cancer and other inflammatory diseases [27].

Many questions about TLS development in oral cancer remain to be elucidated. Ectopic lymphoid formation is a common feature in chronically inflamed tissues and has been found in a number of different diseases at various anatomical sites [11]. After the switch from acute to chronic inflammation, gradual accumulation of lymphocytes as well as promotion of lymphangiogenesis and transformation of blood vessels into lymphocyte-guiding HEVs has been observed [28]. In oral cancer, chronically inflamed tissue precedes most of the tumours [29], providing favourable sites for TLS formation. In our OSCC samples, the TLSs were mainly located in the subepithelial lymphocytic infiltrate close to the tumour front. It would be of great interest to find out why the chronic infiltrate sometimes organizes into these structures. Disclosing the mechanisms that regulate TLS development may give important information on how to improve immune-modulating cancer therapy. Lymphoid neogenesis has been most extensively studied in autoimmune disorders such as rheumatoid arthritis, Sjögrens’ syndrome and Hashimoto’s thyroiditis, where TLSs might contribute to disease progression [11]. In some ectopic GCs, B-cells producing antibodies against self-antigens have been recognized, but data are still sparse [28]. In OSCC, it is not yet clear which antigenic targets the lymphocytes might react to and whether auto-antigens play a role in the induction of TLSs. In terms of viral agents that are linked to human tumours, human papillomavirus (HPV) has become a topic of interest during the last years. While HPV is a known risk factor for oropharyngeal cancer, it probably plays only a minor role in cancers arising in the oral cavity [30]. In the present study, there was no correlation between HPV-status and TLS formation. Although the antigenic stimuli directing TLS formation in OSSC are unknown, it seems likely that the immune-modulating factors that promote TLS development derive from the cancer cells rather than from autoimmunity or infection. Our results show that TLSs are most likely to form in well-differentiated tumours. It has been proposed that tumour growth might be related to stem and amplifying cell patterns, and that dedifferentiation may play a role in the origin of cancer stem cells (CSCs) in OSCC [31]. CSCs are a minority of malignant cells that are thought to be able to attenuate host anti-tumour immune responses [32]. Thus, one could speculate that dedifferentiation makes the tumour cells less antigenic and thereby elicits a milder inflammatory reaction with lower induction of TLSs. Previous studies on lymphoid neogenesis have revealed that clearance of the inflammation-inducing antigen or clinical therapy are able to cause a complete remission of the ectopic lymphoid structure [9]. This might be advantageous in autoimmune diseases to stop aggravation of the disease. However, as TLSs are thought to be conducive for patient survival in OSCC, characterization of stimulating agents might be used therapeutically to promote TLS formation by presentation of the causative agent. Investigation of circulating lymphocytes in blood samples of OSCC patients may provide new insights into the involvement of the host immune reaction in TLS development. A long-lasting chronic inflammation, as in larger tumours, could promote TLS development. In the present study, however, more TLSs were found in smaller tumours, clearly indicating that TLS formation can also take place in the early phases of tumour growth.

## Conclusion

We found TLS formation to be a positive prognostic factor for patients with OSSC when tumours were analysed at multiple levels. Thus, patients with TLS-positive tumours might benefit from more restrictive treatment while a closer follow-up and more aggressive therapy should be considered for patients with TLS-negative tumours. However, before we can envisage TLSs as prognostic factors in individual clinical management of OSCC patients, larger studies on ectopic lymphoid structures are needed. Our study shows that correct assessment of TLS by immunohistochemistry requires careful analyses. When assessing CD20 B-cell staining, both dense and more diffuse aggregates of B-cells should be considered as putative TLSs. We found however, that about a third of the TLS-positive patients were missed when analysing only one level in the tissue block. This may be due to the fact that we selected blocks with representative tumour material rather than the tumour blocks with most intense inflammation. By selecting differently, the chance of discovering TLSs on a single tissue level might have increased. PCR-based approaches, such as combining analyses of a combination of mature FDC markers, HEV markers and TLS associated chemokines such as CCL19, CCL21 and CXCL13 [21], could also decrease the chance of missing TLS-positive tumours. Furthermore, analyses of TLS associated chemokines in serum from cancer patients could be a possible indicator of TLS formation.

The future trend in clinical cancer management points to personalized treatment. The use of biomarkers to guide treatment decisions along with development of immunotherapy may benefit the patient. Thus, understanding TLS formation in OSCC might help to guide targeted anti-cancer therapies and improve the dismal survival rates of patients with oral cancer.

## Abbreviations

OSCCs: Oral squamous cell carcinomas; TILs: Tumour-infiltrating lymphocytes; SLO: Secondary lymphoid organ; TLS: Tertiary lymphoid structure; FDCs: Follicular dendritic cells; HEV: High-endothelial venule; PNAd: Peripheral node addressin; GC: Germinal centre; CSC: Cancer stem cell.

## Competing interests

The authors declare that they have no competing interest.

## Authors’ contributions

AW carried out the manual immunohistochemical staining, participated in interpretation and scoring of the immunohistochrmical stainings and the statistical analyses and drafted the manuscript. OR retrieved the clinical information from patient journals, participated in the statistical analyses and critically reviewed the manuscript. SES participated in interpretations of the immunohistochemical stainings and the statistical analyses, and critically reviewed the manuscript. LUH participated in design of the study and in interpretations of the immunohistochemical stainings, and critically reviewed the manuscript. EHO participated in design of the study, scoring of the immunohistocehemical stainings and helped to draft the manuscript. All authors read and approved the final manuscript.

## Pre-publication history

The pre-publication history for this paper can be accessed here:

http://www.biomedcentral.com/1472-6890/14/38/prepub

## Supplementary Material

Additional file 1: Table S1 Results from multiple level analysis: Univariate Kaplan Meier analysis of 5-year disease-specific death for 80 patients with oral squamous cell carcinoma with various subtypes of tertiary lymphoid structures (TLSs). **Figure S1.** Results: Log minus log plots for proportional hazards checking; (A) T stage; (B) N stage; (C) tertiary lymphoid structure (TLS). Click here for file
