# Supplementary material for: Characterisation and prognostic value of tertiary lymphoid structures in oral squamous cell carcinoma
Source: BMC Clin Pathol. 2014 Aug 23;14:38. doi: 10.1186/1472-6890-14-38 (PMC4148494; doi:10.1186/1472-6890-14-38)
Supplement: Additional file 1: Table S1 — Results from multiple level analysis: Univariate Kaplan Meier analysis of 5-year disease-specific death for 80 patients with oral squamous cell carcinoma with various subtypes of tertiary lymphoid structures (TLSs). Figure S1. Results: Log minus log plots for proportional hazards checking; (A) T stage; (B) N stage; (C) tertiary lymphoid structure (TLS). [file 1472-6890-14-38-S1.docx]

**Additional file**

| **Table S1**  **Results from multiple level analysis: Univariate Kaplan Meier analysis of 5-year disease-specific death for 80 patients with oral squamous cell carcinoma with various subtypes of tertiary lymphoid structures (TLSs).** | | | |
| --- | --- | --- | --- |
| **Patients** | **N=80**  **(no. (%))** | **5-Year death (%)** | **P-value** |
| classical TLS | 5 (6.3) | 0.0 | 0.150 |
| all others | 75 (93.8) | 36.0 |  |
| non-classical TLSs | 8 (10.0) | 25.0 | 0.574 |
| all others | 72 (90.0) | 34.7 |  |
| both classical and non-classical TLS | 4 (5.0) | 0.0 | 0.165 |
| all others | 76 (95.0) | 35.5 |  |

P-values were calculated using the log-rank test.

**Figure S1**


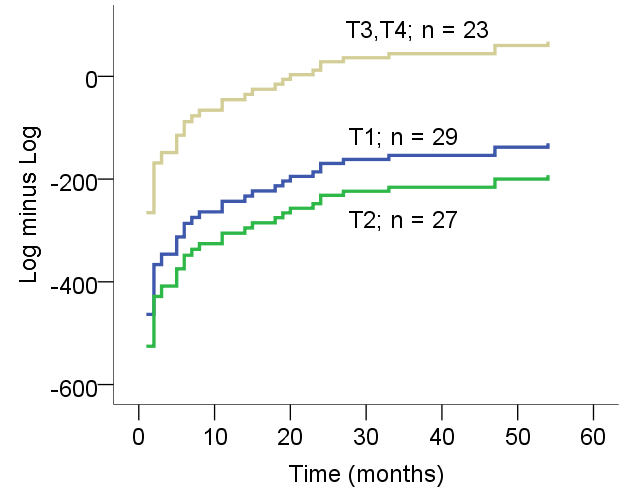
­
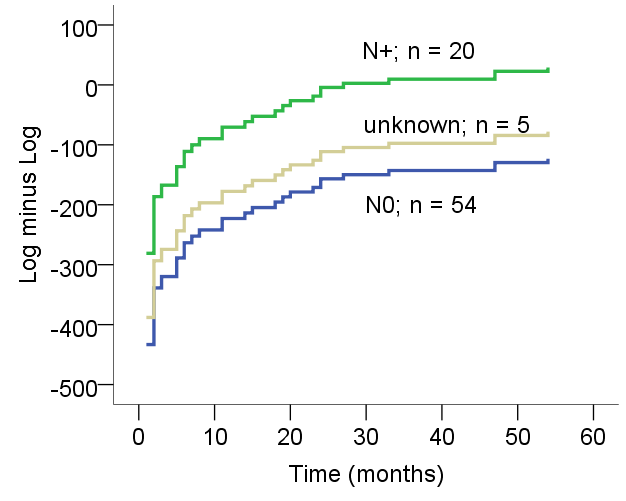

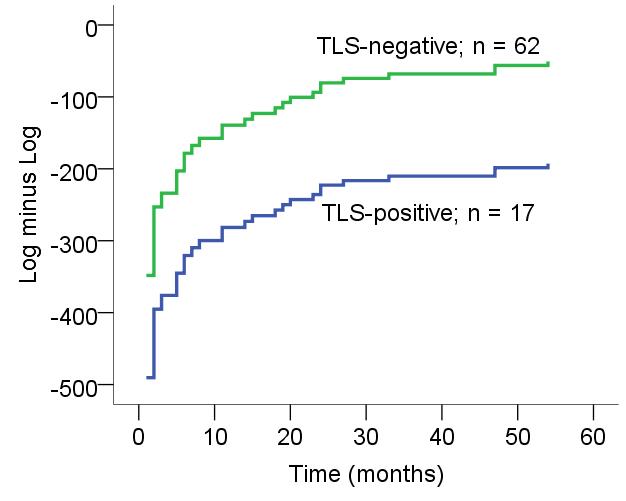


**A**

**C**

**B**

**Results: Log minus log plots for proportional hazards checking; (A) T stage; (B) N stage; (C) tertiary lymphoid structure (TLS).**
